# Research on Trajectory Tracking Control Method for Wheeled Robots Based on Seabed Soft Slopes on GPSO-MPC

**DOI:** 10.3390/s25164882

**Published:** 2025-08-08

**Authors:** Dewei Li, Zizhong Zheng, Zhongjun Ding, Jichao Yang, Lei Yang

**Affiliations:** 1 College of Ocean Science and Engineering, Shandong University of Science and Technology, Qingdao 266590, China; zhengzz0131@163.com (Z.Z.); dzj@ndsc.org.cn (Z.D.); yangjichao@sdust.edu.cn (J.Y.); 2 National Deep Sea Center, Qingdao 266237, China; lyang@ndsc.org.cn

**Keywords:** model predictive control, grey wolf optimizer, particle swarm optimization, wheeled mobile robot, trajectory tracking

## Abstract

With advances in underwater exploration and intelligent ocean technologies, wheeled underwater mobile robots are increasingly used for seabed surveying, engineering, and environmental monitoring. However, complex terrains centered on seabed soft slopes—characterized by wheel slippage due to soil deformability and force imbalance arising from slope variations—pose challenges to the accuracy and robustness of trajectory tracking control systems. Model predictive control (MPC), known for predictive optimization and constraint handling, is commonly used in such tasks. Yet, its performance relies on manually tuned parameters and lacks adaptability to dynamic changes. This study introduces a hybrid grey wolf-particle swarm optimization (GPSO) algorithm, combining the exploratory ability of a grey wolf optimizer with the rapid convergence of particle swarm optimization. The GPSO algorithm adaptively tunes MPC parameters online to improve control. A kinematic model of a four-wheeled differential-drive robot is developed, and an MPC controller using error-state linearization is implemented. GPSO integrates hierarchical leadership and chaotic disturbance strategies to enhance global search and local convergence. Simulation experiments on circular and double-lane-change trajectories show that GPSO-MPC outperforms standard MPC and PSO-MPC in tracking accuracy, heading stability, and control smoothness. The results confirm the improved adaptability and robustness of the proposed method, supporting its effectiveness in dynamic underwater environments.

## 1. Introduction

In recent years, with the rapid development of artificial intelligence, autonomous navigation, and intelligent control technologies, underwater robots, especially remotely operated vehicles (ROVs), have become critical platforms for deep-sea resource exploration, scientific research, and engineering operations due to their strong environmental adaptability and task execution capabilities.

In international research on underwater robotics, significant progress has been made in ROV technology. The U.S. Hydroid’s REMUS 6000 participated in the search for Malaysia Airlines MH370, showcasing strong large-scale deep-sea search capabilities. France’s Ifremer launched the Victor 6000, a 6000 m class ROV with versatile manipulators and precision sampling tools, widely used in hydrothermal vent studies [1]. Britain’s SMD developed the Quantum series heavy-duty ROVs, operating at 6000 m with dual-manipulator collaboration, supporting deep-sea oil and gas development. Japan’s JAMSTEC’s Kaiko ROV reached the 11,000 m Mariana Trench, laying the groundwork for ultra-deep exploration [2]. Domestically, notable progress includes the “Haidou 1” by the Shenyang Institute of Automation, which achieved autonomous navigation and precision sampling at 11,000 m in the Mariana Trench, marking China’s entry into ultra-deep exploration. Shanghai Jiao Tong University’s “Haima” ROV, operating at 4500 m, has completed hundreds of deep-sea samplings, verifying practicality. The “Hailong 2” ROV discovered hydrothermal vents in the eastern Pacific, contributing to deep-sea geology [3]. The 3000 m class “Faxian” ROV has been applied in submarine cable inspection, addressing operational challenges on complex seabeds.

Despite these advancements, trajectory tracking control of ROVs in complex underwater environments remains challenging [4]. Conventional control strategies, such as proportional-integral-derivative (PID) control [5] and sliding mode control (SMC) [6,7], often struggle in narrow passages, sharp turns, or environments with dynamic obstacles. In such situations, accumulated tracking errors can significantly degrade system performance and hinder high-precision control. MPC, known for its receding horizon optimization and explicit constraint-handling capabilities, has emerged as a promising solution for trajectory tracking tasks [8]. However, traditional MPC approaches rely heavily on manually tuned parameters—such as the prediction horizon, control horizon, and weighting matrices—resulting in limited adaptability in complex or highly disturbed scenarios [9]. Enhancing MPC adaptiveness by minimizing manual intervention has thus become a key research challenge in advancing trajectory tracking for WMRs in dynamic underwater environments.

Recent research on adaptive parameter optimization has led to various improved algorithms and hybrid frameworks, which can be broadly categorized into three directions: optimization using intelligent algorithms, prediction via machine learning, and hybrid intelligent optimization strategies [10]. Swarm intelligence algorithms are widely employed due to their parallel search capabilities. For example, Jiao et al. [11] introduced a chaotic PSO (CPSO) with a tent map to enhance diversity, although premature convergence remained an issue. Shi et al. [12] proposed an adaptive-inertia PSO to dynamically adjust learning factors, which effectively reduced tracking errors. Hybrid GA-PSO algorithms [13] demonstrated improved global search performance but incurred high computational costs. Meanwhile, GWO has been applied to dynamically adjust MPC prediction horizons [14]. In the domain of learning-based approaches, deep reinforcement learning (DRL) has shown considerable potential. Lillicrap [15] developed an actor–critic architecture for online parameter tuning in dynamic obstacle environments. Zhang et al. [16] proposed an LSTM-based model to predict optimal parameters from historical data. Hybrid strategies have also gained attention. Zhang et al. [17] combined GWO with simulated annealing to improve convergence, while Tang et al. [18] introduced a fuzzy-PSO system with a rule base for parameter adjustment. Wei et al. [19] proposed a WOA-SA approach that enhanced MPC performance in sharp-turn scenarios.

Despite these advancements, several challenges persist. First, many studies overly simplify the optimization objective by focusing solely on tracking error, often neglecting control smoothness and energy efficiency. Second, high computational complexity hinders real-time applications. Lastly, many methods lack adaptability to dynamic and uncertain underwater environments, limiting their practical effectiveness.

To address these issues, this paper proposes a hybrid intelligent optimization framework—GPSO—that integrates GWO and PSO for adaptive parameter tuning in MPC-based trajectory tracking control. The main contributions of this study are as follows:

1. A nonholonomic kinematic model for the WMR is constructed, and an error-state-based MPC controller is developed. A multi-objective fitness function is designed to optimize tracking accuracy, control smoothness, and energy consumption.
2. A novel GPSO algorithm is introduced by combining the hierarchical search mechanism of GWO with the collaborative learning strategy of PSO. Enhancements include dynamic inertia adjustment, chaotic perturbation, and contraction learning to improve convergence and maintain diversity.
3. The GPSO algorithm is embedded into the MPC framework to enable online adaptive optimization of key parameters (i.e., prediction horizon *Np*, control horizon *Nc*, and weighting matrices *Q*, *R*). This integration significantly enhances the robustness and adaptability of the control system in dynamic and uncertain environments.

## 2. Robot Dynamics and Kinematics Modeling

In the exploitation and research of deep-sea resources, the motion control accuracy of wheeled remotely operated vehicles (ROVs) over complex seabed terrains directly determines operational efficiency, thus possessing significant research value. This chapter focuses on the wheel force characteristics on soft sloped terrains, and systematically constructs and analyzes the kinematic and dynamic models of the robot. For the kinematic aspect, emphasis is placed on tire slip on seabed slopes: through body–terrain coordinate transformations and by introducing parameters such as slip ratio and sideslip angle, a model considering both longitudinal and lateral slips is developed to accurately describe pose variations in complex terrains. For the dynamic aspect, by exploring the wheel–terrain interaction mechanisms in soft sand and combining this with the nonlinear contact behavior of seabed sediments, a model reflecting their interaction mechanism is established. This model reveals the movement characteristics of the robot in complex seabed environments, laying a theoretical foundation for high-precision motion control.

### 2.1. Robot Kinematic Modeling

**Figure 1:**
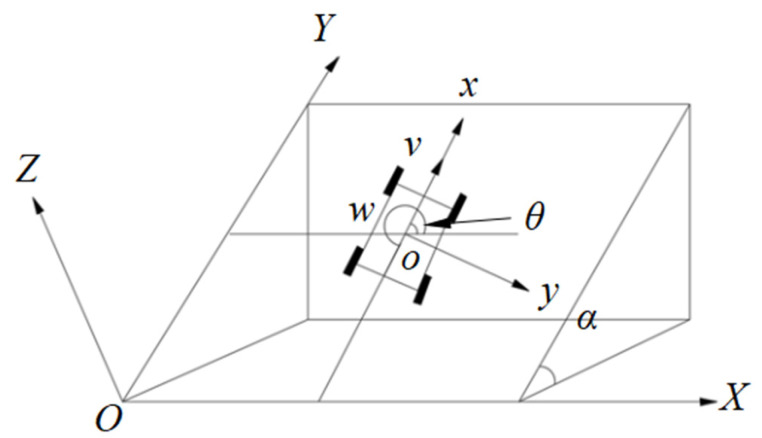
Three-Dimensional Coordinate Relationship Diagram on the Soft Slope.

The research object of this paper is a four-wheeled differential-drive mobile robot, whose three-dimensional coordinate relationship on submarine soft slopes is illustrated in Figure 1. To analyze the motion behavior of the wheeled mobile robot on soft slopes, two spatial coordinate systems are defined as follows: a local coordinate frame (denoted as *oxyz*) is established at the robot’s center of mass, whereas the world inertial coordinate system (*OXYZ*) serves as the global reference frame.

The robot’s pose in the global coordinate system is represented as *q = [x y θ]^T^*, where (*x*,*y*) denotes the position of the robot’s center of mass in the global frame, and *θ* represents the heading angle—defined as the orientation between the robot’s longitudinal axis and the global *X*-axis. *v* denotes the linear velocity along the *x*-axis of the robot’s body-fixed frame, and *w* represents the angular velocity about its center of mass.

**Figure 2:**
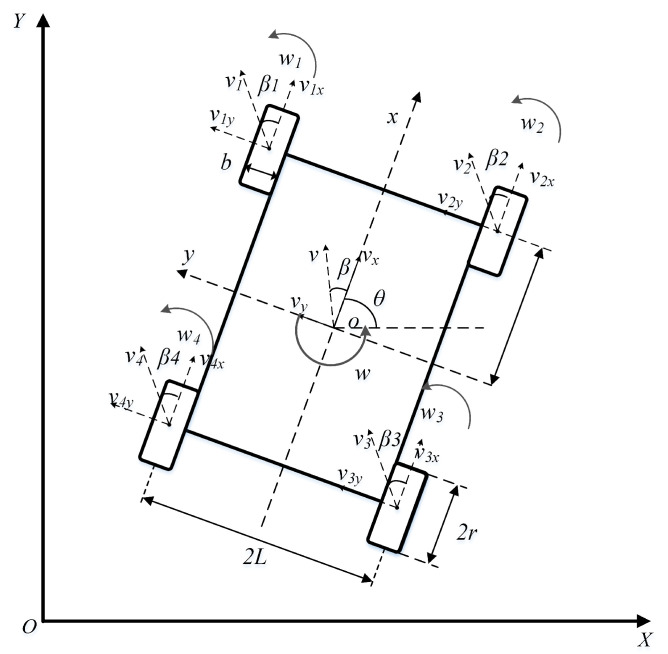
Robot Kinematic Model on Soft Slopes.

Considering the slippage phenomena and slip conditions of each wheel for a four-wheeled robotic vehicle operating on complex soft-sloped terrains of the actual seabed, a simplified kinematic model is constructed, with its schematic diagram shown in Figure 2. Table 1 presents the nomenclature of the robot kinematic model, which explains the symbols and parameters involved in the model. When a wheeled robot slips on a slope, its actual linear velocity *v* can be decomposed into components along the *x*-axis and *y*-axis of the robot’s body frame. The relationship between the linear velocity and its longitudinal/lateral components is expressed as follows:

(1) $$v_{x}=v\cos\beta ,\;v_{y}=v\sin\beta$$

The relationship between the local coordinate frame and the inertial (global) coordinate frame is established, with the corresponding velocity transformation derived as follows [20]:

(2) $$\dot{q}=\left[\begin{array}{c} \dot{x}\\ \dot{y}\\ \dot{\theta } \end{array}\right]=\left[\begin{array}{ccc} \cos\theta & -\sin\theta & 0\\ \sin\theta & \cos\theta & 0\\ 0 & 0 & 1 \end{array}\right]\left[\begin{array}{c} v_{x}\\ v_{y}\\ w \end{array}\right]$$

On soft terrains, variations in the normal load on each wheel lead to corresponding changes in their slip ratios and sideslip angles. For differential-steered robots, the longitudinal velocities of wheels on the same side remain consistent during straight-line travel, whereas a speed difference emerges between the wheels on the two sides during turning. From this, the relationship between wheel velocities and the robot’s traveling speed can be derived as follows:

(3) $$\left\{\begin{array}{l} v_{x}=v_{1}\cos\beta_{1}+Lw=v_{2}\cos\beta_{2}-Lw=v_{3}\cos\beta_{3}-Lw=v_{4}\cos\beta_{4}+Lw\\ v_{y}=v_{1}\sin\beta_{1}-Dw=v_{2}\sin\beta_{2}-Lw=v_{3}\sin\beta_{3}+Lw=v_{4}\sin\beta_{4}+Lw \end{array}\right.$$

The sideslip angle satisfies the small-angle approximation condition:

(4) cosβ≈1, sinβ≈β, tanβ≈β

(5) $$\left\{\begin{array}{l} \beta_{1}=\tan^{-1}(\frac{v\sin\beta +wD}{v\cos\beta -wL})\approx \frac{\dot{y}+wD}{\dot{x}-wL}=\frac{v\beta +wD}{v-wL}\\ \beta_{2}=\tan^{-1}(\frac{v\sin\beta +wD}{v\cos\beta +wL})\approx \frac{\dot{y}+wD}{\dot{x}+wL}=\frac{v\beta +wD}{v+wL}\\ \beta_{3}=\tan^{-1}(\frac{v\sin\beta -wD}{v\cos\beta +wL})\approx \frac{\dot{y}-wD}{\dot{x}+wL}=\frac{v\beta -wD}{v+wL}\\ \beta_{4}=\tan^{-1}(\frac{v\sin\beta -wD}{v\cos\beta -wL})\approx \frac{\dot{y}-wD}{\dot{x}-wL}=\frac{v\beta -wD}{v-wL} \end{array}\right.$$

On soft terrain, relative sliding occurs at the contact interface between the tires and the ground, inducing wheel slippage and consequent partial loss of driving force. To accurately characterize the wheel slippage behavior, the slip coefficients of the four driving wheels are defined as follows:

(6) $$k_{si}=\frac{v_{deal,i}-v_{actual,i}}{v_{deal,i}},\;\left(i=1,2,3,4\right)$$

Further, the kinematic equation can be derived as follows:

(7) $$\dot{q}=\left[\begin{array}{cccc} \frac{\left(1-k_{s1})\cos(\theta +\beta_{1}\right)}{4} & \frac{\left(1-k_{s2})\cos(\theta +\beta_{2}\right)}{4} & \frac{\left(1-k_{s3})\cos(\theta +\beta_{3}\right)}{4} & \frac{\left(1-k_{s4})\cos(\theta +\beta_{4}\right)}{4}\\ \frac{\left(1-k_{s1})\sin(\theta +\beta_{1}\right)}{4} & \frac{\left(1-k_{s2})\sin(\theta +\beta_{2}\right)}{4} & \frac{\left(1-k_{s3})\sin(\theta +\beta_{3}\right)}{4} & \frac{\left(1-k_{s4})\sin(\theta +\beta_{4}\right)}{4}\\ \frac{(1-k_{s1})\cos\beta_{1}}{4L} & -\frac{(1-k_{s2})\cos\beta_{2}}{4L} & -\frac{(1-k_{s3})\cos\beta_{3}}{4L} & \frac{(1-k_{s4})\cos\beta_{4}}{4L} \end{array}\right]\left[\begin{array}{c} v_{1}\\ v_{2}\\ v_{3}\\ v_{4} \end{array}\right]$$

### 2.2. Robot Dynamics Modeling

#### 2.2.1. Analysis of Wheel–Terrain Interaction Forces

**Figure 3:**
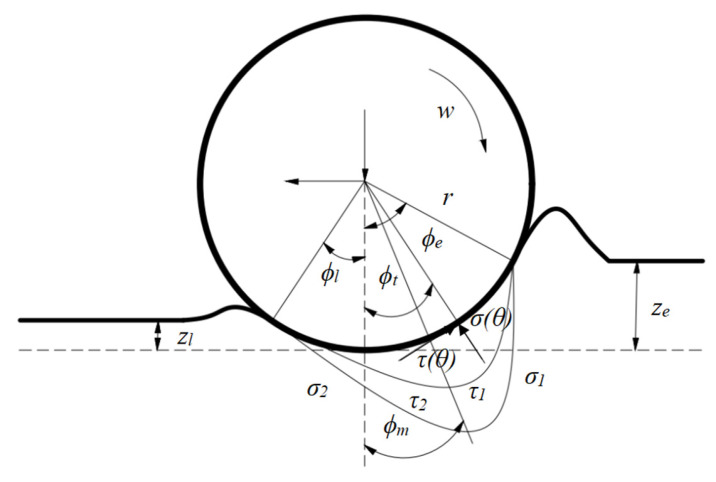
Force Analysis Diagram of Robot–Terrain Interaction.

The movement characteristics of wheeled ROVs in soft seabed sandy environments differ significantly from those on hard, flat surfaces. Due to the distinct physical property of soft soil—its proneness to deformation—phenomena such as longitudinal skidding, lateral wheel sliding, and even wheel subsidence are highly likely to occur. Figure 3 illustrates the wheel–soil interaction force model.

For wheeled robots operating in soft sandy environments, the key parameters characterizing the wheel motion state are as follows: (1) The sideslip angle *β* denotes the angle between the actual motion direction of the wheel and the longitudinal axis of the vehicle body. (2) The wheel–soil contact angle *ϕ_t_* defines the contact range between the wheel and the soil, which influences the distribution of shear forces. It includes the entry angle *ϕ_e_*, exit angle *ϕ_l_*, and maximum stress angle *ϕ_m_*. (3) The slip ratio *s* reflects the deviation between the actual and ideal speeds of the wheel: when *k_si_* > 0, it indicates slipping (actual speed < theoretical speed); when *k_si_* = 0, pure rolling occurs; when *k_si_* < 0, it indicates sliding (actual speed > theoretical speed). The wheel slip speed *v_s_* can be expressed as follows:

(8) $$v_{s}=r\omega -v\cos\phi_{t}=r\omega \left[1-(1-s)\cos\phi_{t}\right]$$

Furthermore, the longitudinal shear displacement at the wheel–soil interface can be derived through integration.

(9) $$j(\phi_{t})=r[\phi_{e}-\phi_{t}-(1-s)(\sin\phi_{e}-\sin\phi_{t})]$$

The tangential shear strength of soil dictates the maximum traction force of the wheel, thus making shear characteristics a critical factor in robot motion. Using the Wong–Reece and Janosi-Hanamoto soil mechanics models, the formulas for calculating normal stress and shear stress at any point within the wheel–soil contact interface are derived as follows:

(10) $$\left\{\begin{array}{l} \sigma (\phi_{t})=(\frac{k_{c}}{b}+k_{\varphi })r^{n}{\left(\cos\phi_{t}-\cos\phi_{e}\right)}^{n}\\ \tau (\phi_{t})=(c+\sigma (\phi_{t})\tan\varphi )(1-e^{-j(\phi_{t})/k_{j}}) \end{array}\right.$$

where *c* represents the cohesion parameter of the soil; *k_j_* denotes the shear deformation amount of the soil; *k_c_* stands for the cohesive deformation modulus; *n* is the sinkage exponent; *φ* is the internal friction angle of the soil; and *k_φ_* represents the frictional deformation modulus. The parameters of the seabed soil referred to above are set out in Table 2. Accordingly, the traction force *F_ti_*, torque of the drive-wheel motor *T_i_*, and wheel normal load *W_i_* can be derived as follows:

(11) $$\left\{\begin{array}{l} W_{i}=rb\left\{\int_{\phi_{l}}^{\phi_{m}}\left[\sigma_{2}(\phi_{t})\cos\phi_{t}+\tau_{2}(\phi_{t})\sin\phi_{t}\right]d\phi_{t}+\int_{\phi_{m}}^{\phi_{e}}\left[\sigma_{1}(\phi_{t})\cos\phi_{t}+\tau_{1}(\phi_{t})\sin\phi_{t}\right]d\phi_{t}\right\}\\ F_{ti}=rb\left\{\int_{\phi_{l}}^{\phi_{m}}\left[\tau_{2}(\phi_{t})\cos\phi_{t}-\sigma_{2}(\phi_{t})\sin\phi_{t}\right]d\phi_{t}+\int_{\phi_{m}}^{\phi_{e}}\left[\tau_{1}(\phi_{t})\cos\phi_{t}-\sigma_{1}(\phi_{t})\sin\phi_{t}\right]d\phi_{t}\right\}\\ T_{i}=r^{2}b\left[\int_{\phi_{l}}^{\phi_{m}}\tau_{2}(\phi_{t})d\phi_{t}+\int_{\phi_{m}}^{\phi_{e}}\tau_{1}(\phi_{t})d\phi_{t}\right] \end{array}\right.$$

#### 2.2.2. Robot Dynamic Modeling on Soft Slopes

**Figure 4:**
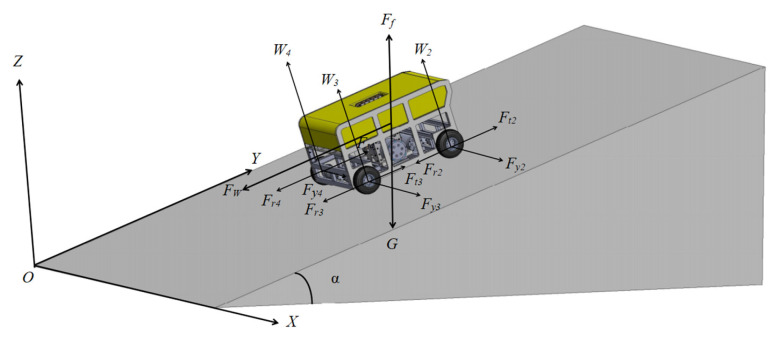
Robot Dynamic Model on Soft Slopes.

When the robot moves on an inclined plane, it is subjected to a vertically upward buoyancy force *F_f_*, a vertically downward gravitational force *G*, and a water resistance force *F_W_* acting opposite to the forward direction. Each wheel experiences a traction force *F_ti_*, a ground resistance force *F_ri_*, a lateral force *F_yi_*, and a normal load *W_i_* perpendicular to the inclined plane. The force diagram of the ROV on the seabed slope is presented in Figure 4, and the nomenclature of robot dynamic model on soft slope in Table 3.

When the robot is situated on slopes with varying angles, the normal loads borne by individual wheels differ. Based on the force and moment equilibrium conditions of the robot, the respective normal loads of the four wheels can be derived as follows:

(12) $$\left\{\begin{array}{l} W_{1}=\frac{G_{z}}{4}-\frac{G_{x}h}{4D}-\frac{G_{y}h}{4L}\\ W_{2}=\frac{G_{z}}{4}-\frac{G_{x}h}{4D}+\frac{G_{y}h}{4L}\\ W_{3}=\frac{G_{z}}{4}+\frac{G_{x}h}{4D}+\frac{G_{y}h}{4L}\\ W_{4}=\frac{G_{z}}{4}+\frac{G_{x}h}{4D}-\frac{G_{y}h}{4L} \end{array}\right.$$

where: $G_{x}=\sin\alpha \sin\theta$, $G_{y}=\sin\alpha \cos\theta$, $G_{z}=\cos\alpha$.

The lateral force of a wheel is jointly influenced by its sideslip angle and normal load. Consequently, an approximately linearized lateral force formula is adopted in this study, where *c_β_* denotes the wheel relative cornering stiffness [21].

(13) $$\left\{\begin{array}{l} F_{y1}=-c_{\beta }W_{1}\beta_{1}=-c_{\beta }W_{1}\frac{v_{y}+Dw}{v_{x}-Lw}\\ F_{y2}=-c_{\beta }W_{2}\beta_{2}=-c_{\beta }W_{2}\frac{v_{y}+Dw}{v_{x}+Lw}\\ F_{y3}=-c_{\beta }W_{3}\beta_{3}=-c_{\beta }W_{3}\frac{v_{y}-Dw}{v_{x}+Lw}\\ F_{y4}=-c_{\beta }W_{4}\beta_{4}=-c_{\beta }W_{4}\frac{v_{y}-Dw}{v_{x}-Lw} \end{array}\right.$$

Using the Newton–Euler system modeling approach, the three-degree-of-freedom dynamic equilibrium equations for the ROV can be established as follows:

(14) $$\left\{\begin{array}{l} m\ddot{X}-m\dot{Y}w=\cos\theta \sum_{i=1}^{4}(F_{ti}-F_{ri})+\sin\theta \sum_{i=1}^{4}F_{yi}-(G-F_{f})\sin\alpha \sin\theta +\cos\theta F_{W}\\ m\ddot{Y}-m\dot{X}w=\sin\theta \sum_{i=1}^{4}\left(F_{ti}-F_{ri}\right)-\cos\theta \sum_{i=1}^{4}F_{yi}-(G-F_{f})\sin\alpha \cos\theta -\sin\theta F_{W}\\ I_{z}\dot{w}=L(F_{DP2}+F_{DP3}-F_{DP1}-F_{DP4})+d(F_{y1}+F_{y2}-F_{y3}-F_{y4}) \end{array}\right.$$

where *F_ri_ = μW_i_*, *μ* is the ground friction coefficient; *I_z_* represents the moment of inertia of the robot. The simplified hydrodynamic drag formula is as follows:

(15) $$\;F_{W}=\frac{1}{2}{\rho C_{d}Av_{w}}^{2}$$

where *ρ* is the seawater density; *C_d_* is the drag coefficient; *A* is the projected area of the robot perpendicular to the direction of motion; and *v_w_* is the speed of the robot relative to the water flow.

The kinematic Equation (7) of the robot is defined as $\dot{q}=S(q)u$. By differentiating this equation and substituting relevant expressions, and then through derivation and simplification, a dynamic model in the form of a matrix equation can be obtained:

(16) $$\hat{M}(q)\dot{\hat{v}}+\hat{V}(q,\dot{q})\hat{v}+{\hat{F}}_{w}(\dot{q})+\hat{G}(q)+{\hat{F}}_{r}(\dot{q})+{\hat{\tau }}_{d}=\hat{B}(q)\tau$$

where: $\hat{v}={\left[v_{1},v_{2},v_{3},v_{4}\right]}^{T}$, M(q) is the inertia force matrix, $\hat{V}(q,\dot{q})$ is the Coriolis force and centrifugal force matrix, ${\hat{F}}_{w}(\dot{q})$ is the water resistance term, $\hat{G}(q)$ is the gravity term, ${\hat{F}}_{r}(\dot{q})$ is the ground resistance term, ${\hat{\tau }}_{d}$ is the unknown disturbance term, $\hat{B}(q)$ is the input transmission matrix, and τ is the motor driving torque.

## 3. Model Predictive Controller Design

Based on the kinematic model established in the preceding section, a discrete linearized prediction model of the robot is constructed, and an MPC-based trajectory tracking controller is designed. The design process includes system linearization, prediction model construction, formulation of the optimization objective function, incorporation of system constraints, and the development of a control input feedback mechanism.

### 3.1. Model Prediction

The nonlinear dynamics model expressed by Equation (16) can be expressed as follows:

(17) $$\dot{q}(t)=f[q(t),u(t)]$$

To improve computational efficiency, a first-order Taylor expansion is performed around the reference trajectory, neglecting higher-order terms. This yields the linearized continuous-time model:

(18) $$\dot{q}=f(q_{t},u_{t})+\frac{\partial f}{\partial q}{|}_{\begin{array}{l} q=q_{t}\\ u=u_{t} \end{array}}(q-q_{t})+\frac{\partial f}{\partial u}{|}_{\begin{array}{l} q=q_{t}\\ u=u_{t} \end{array}}(u-u_{t})$$

By subtracting Equation (18) from Equation (17), the linear error-state model is obtained:

(19) $$\dot{\bar{q}}=A(t)\bar{q}+B(t)\bar{u}$$

The system Jacobian matrix is defined as follows:

(20) $$A(t)=\frac{\partial f}{\partial q}{|}_{\begin{array}{l} q=q_{t}\\ u=u_{t} \end{array}},\;B(t)=\frac{\partial f}{\partial u}{|}_{\begin{array}{l} q=q_{t}\\ u=u_{t} \end{array}}$$

Through Euler discretization with sampling period *T*, the continuous model is converted into a time-varying discrete state-space representation [22]:

(21) $$\bar{q}(k+1)=A_{k,t}\bar{q}(k)+B_{k,t}\bar{u}(k)$$

where $A_{k,t}=I+TA(t)$, $B_{k,t}=I+TB(t)$.

To uniformly incorporate control input increments and state constraints, an extended state vector is introduced:

(22) $$\xi (k|t)=\left[\begin{array}{c} \bar{q}(k|t)\\ \bar{u}(k-1|t) \end{array}\right]$$

By substituting Equation (22) into Equation (21), a new state-space equation is obtained:

(23) $$\left\{\begin{array}{l} \xi (k+1|t)={\bar{A}}_{k,t}\xi (k|t)+{\bar{B}}_{k,t}\Delta u(k|t)\\ \eta (k|t)={\bar{C}}_{k,t}\xi (k|t) \end{array}\right.$$

Given the prediction horizon *Np* and control horizon *Nc*, the predicted state and output at time *k* + *Np* are derived from Equation (22) as follows:

(24) $$\left\{\begin{array}{l} \xi (k+N_{p}|t)={{\bar{A}}_{k,t}}^{N_{p}}\xi (k|t)+{{\bar{A}}_{k,t}}^{N_{p}-1}{\bar{B}}_{k,t}\Delta u(k|t)+\cdot \cdot \cdot +{{\bar{A}}_{k,t}}^{N_{p}-N_{c}-1}{\bar{B}}_{k,t}\Delta u(k+N_{c}|t)\\ \eta (k+N_{p}|t)={\bar{C}}_{k,t}{{\bar{A}}_{k,t}}^{N_{p}}\xi (k|t)+{\bar{C}}_{k,t}{{\bar{A}}_{k,t}}^{N_{p}-1}{\bar{B}}_{k,t}\Delta u(k|t)+\cdot \cdot \cdot +{\bar{C}}_{k,t}{{\bar{A}}_{k,t}}^{N_{p}-N_{c}-1}{\bar{B}}_{k,t}\Delta u(k+N_{c}|t) \end{array}\right.$$

Further derivation yields the predicted output over the entire prediction horizon in compact matrix form:

(25) $$Y(t)=\psi_{t}\xi (k|t)+\Phi_{t}\Delta U(t)$$

where

$$Y(t)=\left[\begin{array}{c} \eta (k+1|t)\\ \eta (k+2|t)\\ \cdot \cdot \cdot \\ \begin{array}{c} \begin{array}{c} \eta (k+N_{c}|t)\\ \cdot \cdot \cdot \end{array}\\ \eta (k+N_{p}|t) \end{array} \end{array}\right],\;\psi_{t}=\left[\begin{array}{c} \begin{array}{c} \begin{array}{c} {\bar{C}}_{k,t}{\bar{A}}_{k,t}\\ {\bar{C}}_{k,t}{{\bar{A}}_{k,t}}^{2} \end{array}\\ \cdot \cdot \cdot \end{array}\\ {\bar{C}}_{k,t}{{\bar{A}}_{k,t}}^{N_{c}}\\ \cdot \cdot \cdot \\ {\bar{C}}_{k,t}{{\bar{A}}_{k,t}}^{N_{p}} \end{array}\right],\;\Delta U(t)=\left[\begin{array}{c} \Delta u(k|t)\\ \Delta u(k+1|t)\\ \cdot \cdot \cdot \\ \Delta u(k+N_{c}|t) \end{array}\right],$$

$$\Phi_{t}=\left[\begin{array}{cccc} {\bar{C}}_{k,t}{\bar{B}}_{k,t} & 0 & 0 & 0\\ {\bar{C}}_{k,t}{\bar{A}}_{k,t}{\bar{B}}_{k,t} & {\bar{C}}_{k,t}{\bar{B}}_{k,t} & 0 & 0\\ \vdots & \vdots & \ddots & \vdots \\ {\bar{C}}_{k,t}{{\bar{A}}_{k,t}}^{N_{c}-1}{\bar{B}}_{k,t} & {\bar{C}}_{k,t}{{\bar{A}}_{k,t}}^{N_{c}-2}{\bar{B}}_{k,t} & \ldots & {\bar{C}}_{k,t}{\bar{B}}_{k,t}\\ {\bar{C}}_{k,t}{{\bar{A}}_{k,t}}^{N_{c}}{\bar{B}}_{k,t} & {\bar{C}}_{k,t}{{\bar{A}}_{k,t}}^{N_{c}-1}{\bar{B}}_{k,t} & \ldots & {\bar{C}}_{k,t}{\bar{A}}_{k,t}{\bar{B}}_{k,t}\\ \vdots & \vdots & \ddots & \vdots \\ {\bar{C}}_{k,t}{{\bar{A}}_{k,t}}^{N_{p}-1}{\bar{B}}_{k,t} & {\bar{C}}_{k,t}{{\bar{A}}_{k,t}}^{N_{p}-2}{\bar{B}}_{k,t} & \ldots & {\bar{C}}_{k,t}{{\bar{A}}_{k,t}}^{N_{p}-N_{c}-1}{\bar{B}}_{k,t} \end{array}\right]$$

### 3.2. Optimization Solution

To enable the controller to perform online optimization and accurately track the reference trajectory, the following objective function is formulated [23]:

(26) $$J(\xi (t),\Delta u(t),\varepsilon )=\sum_{i=1}^{N_{P}}{\left\|\eta (k+i|t)-\eta_{r}(k+i|t)\right\|}_{Q}^{2}+\sum_{j=1}^{N_{c}-1}||\Delta u(k+j|t){||}_{R}^{2}+\rho \varepsilon^{2}$$

A deviation term *E*(*t*) is introduced to facilitate the solution of the objective function:

(27) $$E(t)=\psi_{t}\xi (k|t)-Y_{r}(t)$$

The objective function is then reformulated into a standard quadratic programming (QP) form [24]:

(28) $$J(\xi (t),\Delta u(t),\varepsilon )=\frac{1}{2}x^{T}H_{t}x+f^{T}x+d_{t}$$

where

$$x=\left[\begin{array}{c} \Delta U(t)\\ \varepsilon \end{array}\right],\;H_{t}=\left[\begin{array}{cc} 2{\Phi_{t}}^{T}Q\Phi_{t}+2R & 0\\ 0 & 2\rho \end{array}\right],\;f^{T}=\left[\begin{array}{c} 2E{\left(t\right)}^{T}Q\Phi_{t}\\ 0 \end{array}\right],\;d_{t}=E{\left(t\right)}^{T}QE(t)$$

System constraints are defined for state variables, control inputs, and output variables as follows:

(29) $$\left\{\begin{array}{l} \Delta U_{\min}\leq \Delta U(t)\leq \Delta U_{\max}\\ U_{\min}\leq A\Delta U(t)+\Delta U(t)\leq U_{\max}\\ Y_{\min}\leq Y(t)\leq Y_{\max}\\ {Y_{sc,}}_{\min}-\varepsilon \leq Y(t)\leq Y_{sc,\max}+\varepsilon \varepsilon \geq 0 \end{array}\right.$$

where $U_{\max}$ and $U_{\min}$ denote the upper and lower bounds of the control inputs; $\Delta U_{\max}$ and $\Delta U_{\min}$ represent the allowable limits for control input increments; $Y_{sc,\max}$ and ${Y_{sc,}}_{\min}$ specify the soft-constrained output bounds; ε is the slack variable introduced to relax the output constraints.

### 3.3. Feedback Mechanism

At each sampling instant, the optimal control sequence is computed by solving the QP problem.

(30) $$\Delta U_{t}^{*}={\left[\Delta u_{t}^{*},\Delta u_{t+1}^{*},\ldots ,\Delta u_{t+N_{c}-1}^{*}\right]}^{T}$$

The first control increment at the current time step is applied and combined with the previous control input to update the current control command.

(31) $$u(t)=u(t-1)+\Delta {u_{t}}^{*}$$

The controller then proceeds to the next sampling period, repeating the above optimization process to achieve real-time closed-loop control.

## 4. Design of Improved Particle Swarm Optimization Algorithm

Although MPC offers strong path-tracking performance, its effectiveness is highly sensitive to the selection of control parameters, including the prediction horizon *Np*, control horizon *Nc*, and weighting matrices Q R. Traditional fixed-parameter tuning approaches rely heavily on empirical experience, lack a systematic framework, and show limited adaptability to dynamic changes across different trajectory scenarios. To improve the adaptability and performance of the MPC controller, this section presents an enhanced PSO algorithm for adaptive tuning of MPC parameters.

The PSO algorithm, originally proposed by Kennedy and Eberhart in 1995, is a stochastic optimization method inspired by swarm intelligence theory [25]. The algorithm simulates the social behavior observed in bird flocking or fish schooling to iteratively search for optimal solutions in a given problem space.

Within an *N*-dimensional solution space and a swarm population of size *M*, the current position and velocity of the *i*-th particle are defined as follows:

(32) $$X_{i}=\left(X_{i1},X_{i2},\ldots ,X_{iN}\right),V_{i}=\left(V_{i1},V_{i2},\ldots ,V_{iN}\right)$$

The velocity and position of each particle are updated using the following equations:

(33) $$\left\{\begin{array}{l} V_{in}^{k+1}=\omega V_{in}^{k}+C_{1}R_{1}(P_{Best\_in}^{k}-X_{in}^{k})+C_{2}R_{2}(G_{Best\_in}^{k}-X_{in}^{k})\\ X_{in}^{k+1}=X_{in}^{k}+V_{in}^{k+1} \end{array}\right.$$

where k denotes the current number of iterations; ω is the inertia weight factor; $C_{1}$ and $C_{2}$ are the cognitive and social learning coefficients, respectively; $R_{1}$ and $R_{2}$ are uniformly distributed random numbers in the range [0, 1]; $P_{Best\_in}$ in represents the personal best position of the *i*-th particle; $G_{Best\_in}$ in denotes the global best position identified by the entire swarm.

In conventional PSO algorithms, the inertia factor ω and learning coefficients $C_{1}$ and $C_{2}$ are typically fixed, which limits the algorithm’s ability to balance global exploration and local exploitation. To overcome this limitation, a fitness-value-based adaptive inertia weight adjustment mechanism is proposed in this study.

(34) $$\omega_{i}=\left\{\begin{array}{cc} \omega_{\min}+\frac{\left(f_{i}-f_{\min})(\omega_{max}-\omega_{min}\right)}{f_{avg}-f_{\min}}, & f_{i}\leq f_{avg}\\ \omega_{max}, & f_{i}>f_{avg} \end{array}\right.$$

where $f_{i}$ denotes the current fitness value of the *i*-th particle; $f_{avg}$ represents the average fitness value of the swarm; and $f_{\min}$ indicates the minimum fitness value within the current population.

Compared to conventional methods, the proposed approach adaptively adjusts the inertia weight based on the fitness level of each particle relative to the overall population. This mechanism encourages particles with lower fitness to enhance global exploration, while guiding those with higher fitness to improve local convergence accuracy, thereby accelerating convergence speed and improving optimization precision.

Furthermore, to overcome limitations associated with fixed learning factors, this study introduces a dynamic adjustment method incorporating chaotic perturbation, building upon the approach in Reference [26]. Specifically, the learning factors $C_{1}$, $C_{2}$ are perturbed using chaotic sequences generated through logistic mapping, ensuring smooth transitions during iterations and reducing search instability caused by abrupt parameter changes.

(35) $$\left\{\begin{array}{l} C_{1}(k+1)=C_{1}(k)+\chi (k)\\ C_{2}(k+1)=C_{2}(k)+\gamma (k)\\ \chi (k)=-\gamma (k)\\ \gamma (k)=\eta (k)\cdot [1+0.1\cdot z_{k}]\\ \begin{array}{cc} z_{k+1}=4z_{k}(1-z_{k}) & z_{0}=1 \end{array} \end{array}\right.$$

(36) $$\eta (k)=\left\{\begin{array}{cc} 0.05\cdot (1-\frac{k}{0.2K})+0.02\cdot \frac{k}{0.2K} & k\leq 0.2K\\ 0.02\cdot (1-\frac{k-0.2K}{0.15K})-0.035\cdot \frac{k-0.2K}{0.15K} & 0.2K\leq k\leq 0.35K\\ -0.035\cdot (1-\frac{k-0.35K}{0.4K})-0.0015\cdot \frac{k-0.35K}{0.4K} & k\geq 0.35K \end{array}\right.$$

where $z_{k}$ denotes the chaotic sequence value generated by the logistic map (with initial value $z_{0}$=1); η(k) represents the iteration-dependent control factor function, where k is the current iteration index and K is the maximum number of iterations.

A contraction factor κ is introduced to regulate the magnitude of the velocity update:

(37) $$\kappa =\frac{2}{\left|2-\phi -\sqrt{\phi^{2}-4\phi }\right|},\phi =C_{1}+C_{2}>4$$

The velocity and position update equations for the enhanced PSO algorithm are formulated as follows, incorporating adaptive inertia weight, dynamic learning factors, and a contraction–expansion coefficient:

(38) $$\left\{\begin{array}{l} V_{in}^{k+1}=\kappa (\omega_{i}V_{in}^{k}+C_{1}(K)R_{1}(P_{Best\_in}^{k}-X_{in}^{k})+C_{2}(K)R_{2}(g_{Best\_in}^{k}-X_{in}^{k}))\\ X_{in}^{k+1}=X_{in}^{k}+V_{in}^{k+1} \end{array}\right.$$

The MPC controller parameter vector to be optimized is defined as follows:

(39) $$\phi =\left[N_{p},N_{c},q_{1},q_{2},q_{3},r_{1},r_{2}\right]$$

where $N_{p}$ is the prediction horizon; $N_{c}$ is the control horizon; $q_{1}$, $q_{2}$, and $q_{3}$ are the weighting coefficients for lateral error, longitudinal error, and yaw error in the weighting matrix Q; and $r_{1}$, $r_{2}$ are the weighting coefficients for linear and angular velocities in the weighting matrix R.

To enhance the evaluation capability of PSO in MPC parameter optimization, a multi-objective fitness function is designed to guide particles toward optimal parameter combinations.

(40) $$fitness(\phi )=\lambda_{1}\cdot IAE_{e_{y}}+\lambda_{2}\cdot IAE_{e_{x}}+\lambda_{3}\cdot IAE_{e_{\theta }}+\lambda_{3}\cdot TV_{u}+\lambda_{4}\cdot P_{vio}$$

where $IAE_{e_{y}}$ is the integral of the absolute lateral error; $IAE_{e_{x}}$ is the integral of the absolute longitudinal error; $IAE_{e_{\theta }}$ is the integral of the absolute yaw error; $TV_{u}=\sum_{k=1}^{T-1}\left(|v_{k}-v_{k-1}\right)|+|\omega_{k}-\omega_{k-1}|)$ denotes the total variation of control inputs; and $P_{vio}$ represents the soft-constraint penalty term. The penalty term is defined as follows:

(41) $$P_{vio}=\sum_{k=1}^{T}\left[max(0,|e_{y}(k)|-e_{y,max})+max(0,|e_{\theta }(k)|-e_{\theta ,max})+max(0,|v(k)|-v_{max})+max(0,|w(k)|-w_{max})\right]$$

where $e_{y,max}$, $e_{\theta ,max}$, and $v_{max}$, $w_{max}$ denote the maximum allowable errors and control input limits, respectively. The weighting coefficients $\lambda_{1}$ to $\lambda_{4}$ in the fitness function require empirical tuning based on trajectory characteristics to balance performance metrics.

## 5. Design and Optimization of the GPSO-MPC Algorithm

**Figure 5:**
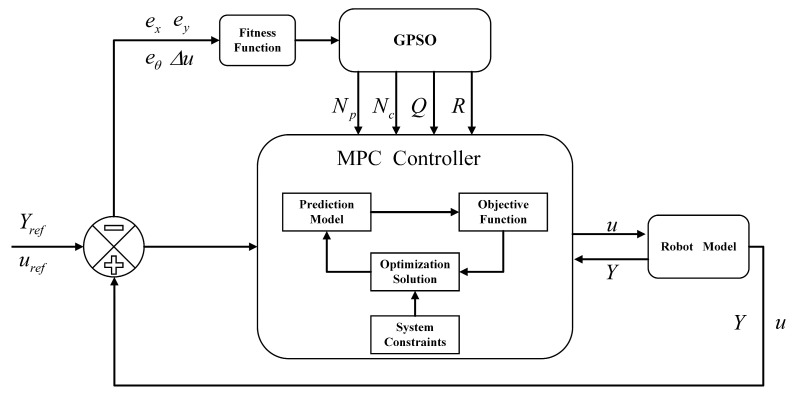
GPSO-MPC Adaptive Trajectory Tracking Control Framework.

Although the improved PSO algorithm demonstrates strong search capability and computational efficiency, it still suffers from premature convergence, entrapment in local optima, and population diversity degradation in complex parameter spaces. These limitations reduce its effectiveness in solving nonlinear, multimodal optimization problems. To overcome them, this section introduces a hybrid GPSO strategy that integrates GWO with PSO. By combining their complementary strengths, the proposed approach improves global optimization performance and enhances robustness in tuning MPC controller parameters. Figure 5 presents the control block diagram of the proposed GPSO-MPC system.

Where $Y_{ref}$ and Y denote the reference and actual state vectors, respectively; $u_{ref}$ and u represent the reference and actual control inputs; $e_{x}$, $e_{y}$, $e_{\theta }$, and Δu indicate the longitudinal error, lateral error, heading angle error, and control input increment, respectively.

GWO, proposed by Mirjalili et al. in 2014, is a metaheuristic swarm intelligence algorithm inspired by the social hierarchy and cooperative hunting strategies of grey wolf packs [27]. The population is divided into four hierarchical roles: α wolves (dominant leaders guiding the pack), β wolves (secondary decision-makers), δ wolves (tertiary scouts), and ε wolves (followers that help maintain population diversity). The population evolves toward optimal solutions by cooperatively tracking the positions of the three dominant wolves (α, β, δ), ensuring a balanced exploration–exploitation trade-off. The position update mechanism is mathematically formulated as follows:

(42) $$\left\{\begin{array}{l} D_{\alpha }(k)=|B_{1}\cdot X_{\alpha }(k)-X_{i}(k)|,X_{1}(k+1)=X_{\alpha }(k)-A_{1}\cdot D_{\alpha }(k)\\ D_{\beta }(k)=|B_{2}\cdot X_{\beta }(k)-X_{i}(k)|,X_{2}(k+1)=X_{\beta }(k)-A_{2}\cdot D_{\beta }(k)\\ D_{\delta }(k)=|B_{3}\cdot X_{\delta }(k)-X_{i}(k)|,X_{3}(k+1)=X_{\delta }(k)-A_{3}\cdot D_{\delta }(k) \end{array}\right.$$

The final position is computed as the weighted average of the three position updates derived from the α, β, and δ wolves’ guidance strategies.

(43) $$X_{i}(k+1)=\frac{X_{1}+X_{2}+X_{3}}{3}$$

where k is the current iteration index; $X_{\alpha }(k)$, $X_{\beta }(k)$, and $X_{\delta }(k)$ denote the position vectors of α, β, and δ wolves at iteration k, respectively; $X_{1}(k+1)$, $X_{2}(k+1)$, and $X_{3}(k+1)$ represent the guided positions of grey wolves by α, β, and δ wolves at iteration k+1, with the final updated position $X_{i}(k+1)$ obtained by averaging these three positions. Vectors A and B are control parameters regulating search scope and direction, calculated as follows:

(44) $$A=2ar_{1}-a,B=2r_{2}$$

where $r_{1}$ and $r_{2}$ are random numbers uniformly distributed in the interval [0, 1], and a is the convergence factor. In this study, an exponentially decaying convergence factor is designed to balance global exploration and local exploitation throughout the iteration process.

(45) $$a=2-\frac{e^{\frac{1}{k}}}{2e}$$

Furthermore, based on Equation (35), a new improvement incorporating learning factor $C_{3}$ is introduced.

(46) $$\left\{\begin{array}{l} C_{3}(k+1)=C_{3}(k)+\beta (k)\\ \chi (k)=-\gamma (k)-\beta (k)\\ \beta (k)=\eta (k)\cdot [1-0.05\cdot z_{k}] \end{array}\right.$$

The formulation of the hybrid velocity–position update is provided in Equation (38):

(47) $$\left\{\begin{array}{l} V_{in}^{k+1}=\kappa (\omega_{i}V_{in}^{k}+C_{1}(K)R_{1}(X_{1}^{k+1}-X_{in}^{k})+C_{2}(K)R_{2}(X_{2}^{k+1}-X_{in}^{k})+C_{3}(K)R_{3}(X_{3}^{k+1}-X_{in}^{k}))\\ X_{in}^{k+1}=X_{in}^{k}+V_{in}^{k+1} \end{array}\right.$$

**Figure 6:**
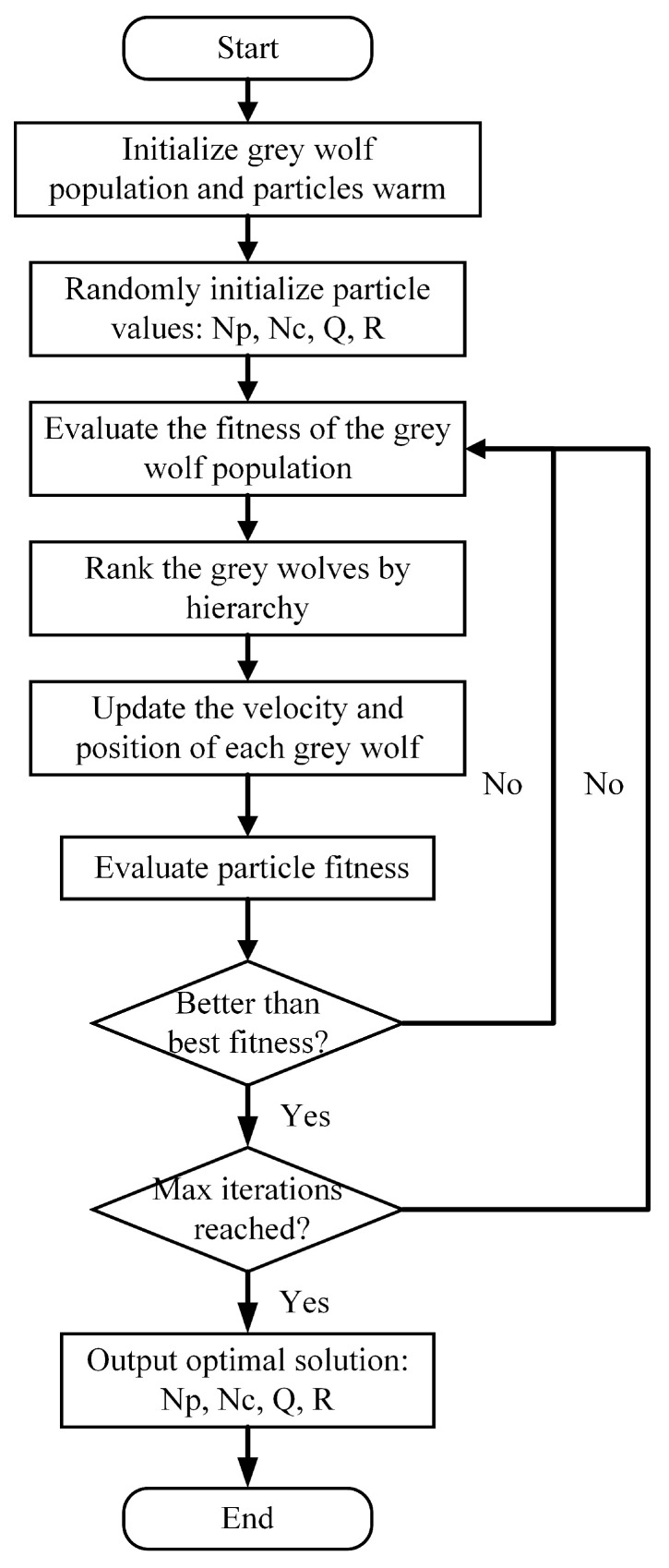
Flowchart of the GPSO hybrid optimization algorithm.

The hybrid algorithm operates on two fundamental mechanisms: (1) utilizing the directional guidance provided by the α, β, and δ leaders in GWO to steer the population toward optimal regions within the search space; and (2) retaining the PSO velocity update mechanism, which considers both personal best and global best positions to enhance dynamic adaptability and solution stability. Figure 6 presents the comprehensive workflow of this integrated GPSO approach. Consequently, the enhanced PSO velocity–position update strategy is effectively incorporated into the GWO architecture.

## 6. Experiment and Result Analysis

To comprehensively evaluate the control performance of the proposed GPSO-MPC path tracking method under complex trajectories and disturbed environments, we developed a high-fidelity simulation model of a four-wheeled, differentially driven underwater mobile robot using the MATLAB platform ((Version 2021)). Systematic experiments were conducted to assess tracking performance across representative scenarios. Comparative analyses were performed among three control strategies—conventional MPC, PSO-MPC, and the proposed GPSO-MPC—with particular focus on tracking accuracy, attitude stability, and control robustness. The key simulation parameters for robot technical parameters and the PSO optimization are summarized in Table 4 and Table 5, respectively.

### 6.1. Circular Trajectory Tracking Experiment

To validate tracking performance, a circular reference trajectory with a radius of *R* = 5 m was designed. The desired linear velocity was set to $V_{ref}$ = 1.5 m/s, corresponding to an angular velocity of $W_{ref}$ = 0.3 rad/s. The parametric equations of the trajectory are given as follows:

(48) $$\left\{\begin{array}{l} x_{r}(t)=5\cos(0.3t)-5\\ y_{r}(t)=5\sin(0.3t) \end{array}\right.t\in [0,\frac{2\pi }{0.3}]$$

In the circular trajectory, the designed sideslip and longitudinal slip are as follows:

(49) $$\left\{\begin{array}{l} v_{y}=0.235\sin(0.4t)\\ k_{si}=0.08\sin(0.25t)+0.08\cos(0.25t) \end{array}\right.$$

**Figure 7:**
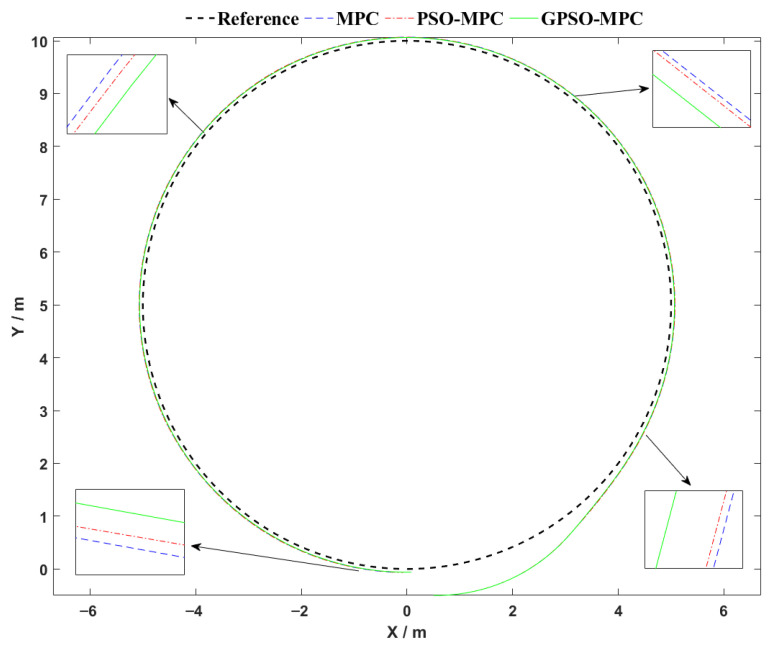
Comparative analysis of circular trajectory tracking performance.

**Figure 8:**
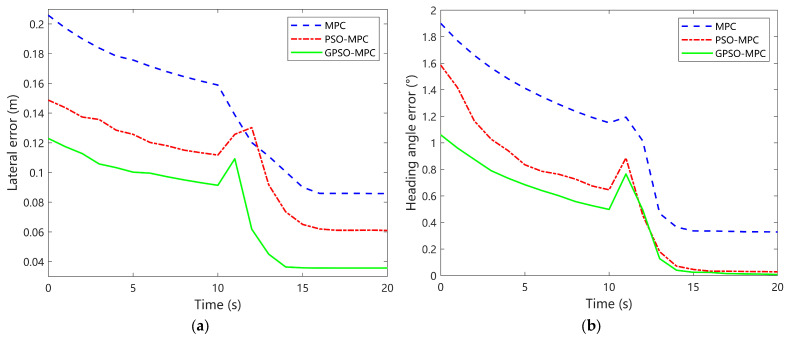
Comparative analysis of tracking errors for circular trajectories. (**a**) Lateral position error; (**b**) Heading angle error.

**Figure 9:**
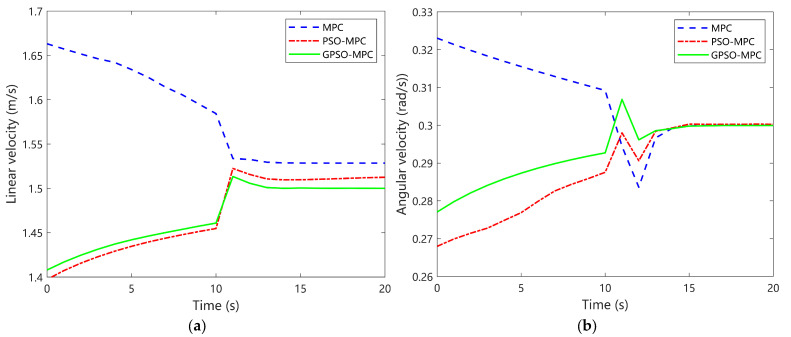
Comparative analysis of tracking output control for circular trajectories. (**a**) Linear velocity; (**b**) Angular velocity.

The robot is initialized at ${\left[x(0),y(0),\theta (0)\right]}^{T}={\left[0.5,-0.5,0\right]}^{T}$, introducing a reasonable initial deviation from the reference path. The controller’s linear velocity was constrained within v∈[0,1.6] m/s, and the angular velocity was limited to w∈[−0.4,0.4] rad/s. To prevent abrupt control fluctuations, rate-of-change constraints were applied: linear velocity variation was limited to Δv≤0.15 m/s, and angular velocity variation was restricted to Δw≤0.1 rad/s. The simulation results corresponding to these conditions are illustrated in Figure 7, Figure 8 and Figure 9.

The circular trajectory tracking results, shown in Figure 7, demonstrate clear performance differences among the three control strategies. The proposed GPSO-MPC controller achieves the highest tracking precision and overall stability throughout the trajectory. Its path nearly aligns perfectly with the reference trajectory, maintaining smooth transitions even in high-curvature regions, thereby demonstrating strong adaptability to dynamic conditions. The PSO-MPC controller performs well in straight-line segments but exhibits noticeable deviation in curved regions. In contrast, the conventional MPC controller shows the largest tracking errors, particularly in areas of high curvature, indicating limited responsiveness to changing path geometries. Overall, the GPSO-MPC strategy outperforms the others in terms of global tracking accuracy, adaptability to curvature, and control smoothness, highlighting its improved robustness and suitability for dynamically varying environments.

Figure 8a illustrates the temporal evolution of lateral position errors for the three control strategies during the circular trajectory tracking task. The MPC controller initially exhibits the largest error, peaking at approximately 0.21 m, and shows a slow rate of error reduction, requiring around 16 s to decrease the error to 0.09 m. The PSO-MPC strategy demonstrates a slight improvement, with the error stabilizing near 0.07 m after approximately 13 s, indicating faster convergence than MPC. In contrast, GPSO-MPC consistently achieves the smallest lateral error, maintaining a maximum deviation below 0.13 m and rapidly converging to about 0.04 m within 13 s—a 23% improvement in steady-state error compared to both MPC and PSO-MPC. In terms of convergence speed, GPSO-MPC achieves a 22% reduction in the time required to reach steady-state error compared to MPC. Overall, GPSO-MPC outperforms the other two methods in lateral error reduction, convergence speed, and steady-state accuracy, particularly excelling in disturbance rejection and recovery from abrupt fluctuations.

Figure 8b shows the temporal variation of heading angle errors for the three strategies. While all methods progressively reduce the initial error to a stable level near zero, they differ significantly in dynamic response. The conventional MPC controller has the largest initial error (around 1.9°) but exhibits more pronounced oscillations later, with sustained fluctuations even at 20 s (steady-state error around 0.35°). The PSO-MPC controller shows a moderate initial error (around 1.6°) but converges faster and stabilizes earlier, with steady-state error near 0.05° at 20 s. In contrast, GPSO-MPC, despite a smaller initial deviation (around 1.1°), demonstrates the fastest response, reducing the heading angle error to a smaller range within 15 s and maintaining a smooth, stable error profile (nearly 0° at 20 s). In summary, MPC struggles with long-term stability despite high initial error amplitude, PSO-MPC balances smoothness and convergence, and GPSO-MPC excels in convergence speed and steady-state precision, demonstrating superior robustness and control regulation in heading performance.

Figure 9a shows the linear velocity profiles of the three control strategies. Although all controllers generally maintain velocities near the reference value of 1.5 m/s, notable differences emerge in their transient responses and adjustment behaviors. The MPC controller produces a relatively smooth velocity curve with minimal fluctuations throughout the tracking process, maintaining values mostly between 1.63 m/s and 1.66 m/s initially, and then decreasing to stabilize near 1.53–1.54 m/s, slightly above the reference. The PSO-MPC initially has a velocity starting from approximately 1.40 m/s at the start, rising to about 1.45 m/s before 10 s, and then converging smoothly to a steady-state value near 1.52 m/s. In contrast, the GPSO-MPC shows a noticeable increase in velocity between 10 and 12 s, with the velocity rising from around 1.45 m/s to about 1.52 m/s, and then a rapid decline and stabilization around 1.50 m/s to 1.51 m/s, closest to the reference in steady state. In summary, although GPSO-MPC introduces some transient variation, it significantly improves responsiveness and adaptability to dynamic conditions, achieving faster adjustment and better tracking precision with minimal steady-state deviation from the target velocity.

Figure 9b shows the temporal evolution of angular velocity outputs for the three control strategies. The MPC controller maintains a relatively high angular velocity in the initial phase, remaining around 0.325 rad/s, and displays a pronounced spike near 10 s, followed by a sharp drop below 0.285 rad/s, indicating limited ability to handle rapid variations. The PSO-MPC controller exhibits a smoother ramp-up from approximately 0.27 rad/s to just below 0.30 rad/s, though it still shows noticeable fluctuations during adjustment. In contrast, the GPSO-MPC controller demonstrates superior responsiveness and regulation under time-varying conditions. Although a brief disturbance appears around 10 s, the angular velocity rapidly converges to a steady-state value very close to 0.30 rad/s. Overall, GPSO-MPC not only adapts more quickly to curvature changes and suppresses oscillations more effectively, but also enables faster convergence to a stable angular velocity.

Table 6 presents the tracking performance under circular trajectory conditions. The GPSO-MPC controller achieves a mean lateral error of 0.075793 m, reducing the error by 45.97% compared to MPC’s 0.14028 m, and lowers the RMSE lateral error by 43.63% to 0.08263 m. For the mean heading angle error, GPSO-MPC reaches 0.45022°, achieving a 23.52% reduction relative to PSO-MPC’s 0.58865°. The RMSE heading angle error of GPSO-MPC drops by 24.63% compared to PSO-MPC’s 0.76093°, reaching 0.57349°. In velocity control, GPSO-MPC maintains a mean linear speed of 1.469 m/s—closer to the 1.5 m/s target than PSO-MPC and reducing deviation by 7.13% compared to MPC’s 1.5817 m/s. Additionally, GPSO-MPC achieves a mean angular velocity of 0.29288 rad/s, only 2.37% below the desired 0.3 rad/s. Overall, GPSO-MPC demonstrates substantial improvements in lateral tracking accuracy and velocity stability, confirming its advantage for precise path tracking on curved trajectories.

### 6.2. Double-Lane Change Trajectory Tracking Experiment

The reference trajectory is defined with a constant linear velocity of $V_{ref}$ = 2 m/s and an angular velocity of $W_{ref}$ = 0 rad/s. The parametric representation of the reference path is formulated as follows:

(50) $$\left\{\begin{array}{c} \begin{array}{c} x_{r}(t)=2t\\ y_{r}(t)=\frac{40}{2}(1+\tanh(z_{1}(t)))-\frac{58}{2}(1+\tanh(z_{2}(t))) \end{array}\\ \theta_{r}(t)=\arctan\left(\left.40\cdot {\left(\mathrm{sech}(z_{1}(t))\right)}^{2}\cdot (\frac{0.98}{8.4})-58\cdot {\left(\mathrm{sech}(z_{2}(t))\right)}^{2}\cdot (\frac{0.98}{16.6})\right)\right.\;\;\;\;\;\;t\in [0,60]\\ z_{1}(t)=\frac{0.98}{4.2}(2t-9.5)-\frac{0.98}{2}\\ z_{2}(t)=\frac{0.98}{8.3}(2t-22)-\frac{0.98}{2} \end{array}\right.$$

In the Double-Lane Change trajectory, the designed sideslip, and longitudinal slip are as follows:

(51) vy=[0.15rect(t,4,12)+0.2rect(t,21,31)]sin(0.4t)ksi=[0.06e−0.2t−5+0.08e−0.2t−23]sin(0.3t)

**Figure 10:**
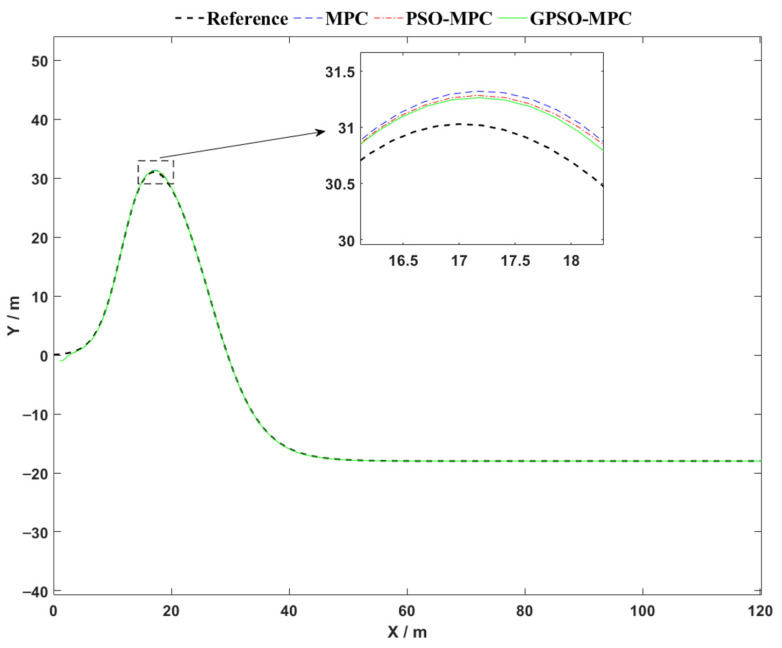
Comparative analysis of trajectory tracking performance on Double-Lane Change.

**Figure 11:**
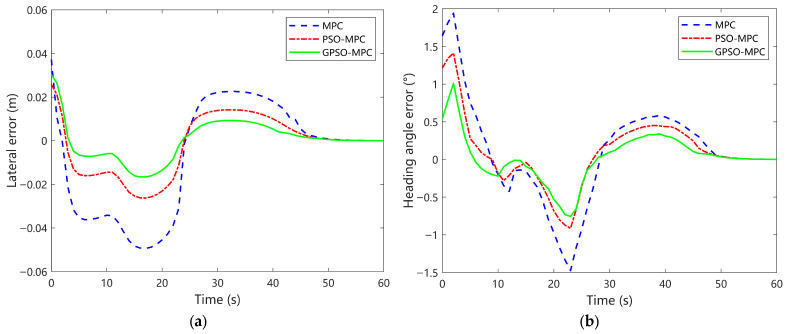
Comparative analysis of tracking errors for Double-Lane Change trajectories. (**a**) Lateral position error; (**b**) Heading angle error.

**Figure 12:**
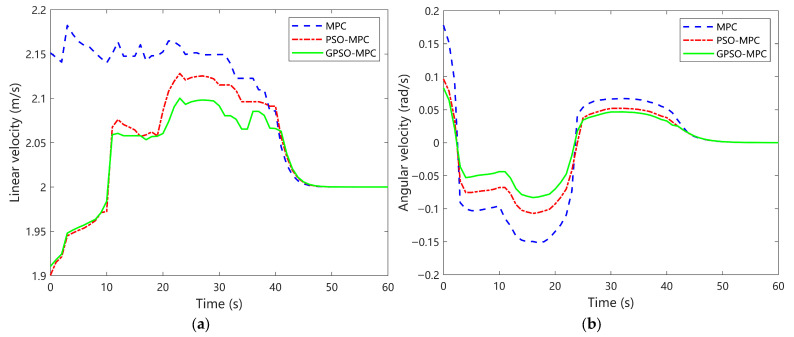
Comparative analysis of tracking output control for Double-Lane Change trajectories. (**a**) Linear velocity; (**b**) Angular velocity.

The experimental setup initializes the robot’s state at ${\left[x(0),y(0),\theta (0)\right]}^{T}={\left[1,-1,0\right]}^{T}$, deliberately introducing a reasonable initial deviation from the reference trajectory to assess system robustness. The controller operates within constrained velocity limits, with linear velocity limited to v∈[0,2.2] m/s and angular velocity to w∈[−0.2,0.2] rad/s. Rate constraints are applied to ensure smooth actuation, limiting linear acceleration to Δv≤0.3 m/s and angular acceleration to Δw≤0.15 rad/s. The simulation results corresponding to these settings are illustrated in Figure 10, Figure 11 and Figure 12.

The tracking performance of the three control strategies along a double-lane-shift trajectory is illustrated in Figure 10. While all methods are generally capable of following the reference path, noticeable differences in local tracking accuracy are observed. In the magnified view, the trajectory produced by the proposed GPSO-MPC controller adheres more closely to the reference path, exhibiting the smallest tracking deviations. In contrast, both the conventional MPC and PSO-MPC approaches show slight misalignments and delayed responses, particularly in high-curvature transition regions. These findings suggest that the GPSO-MPC controller provides superior global optimization capability in parameter tuning, allowing it to more effectively handle rapid nonlinear variations in trajectory geometry and deliver improved control precision and robustness—especially in segments with pronounced curvature changes.

Figure 11 illustrates the temporal variation of tracking errors for the three control strategies during the double-lane-shift trajectory tracking task. Figure 11a shows the lateral tracking errors along the trajectory, which include two sharp turns at approximately 5 s and 18.5 s. The conventional MPC controller exhibits noticeable error fluctuations at both turns, with peak deviations approaching 0.05 m. Although the PSO-MPC method achieves moderate improvement at the first turning segment—maintaining errors within 0.025 m—it still experiences considerable disturbances during the second turn. In contrast, the proposed GPSO-MPC controller demonstrates rapid error convergence in the initial phase, reducing lateral deviations to within 0 m in approximately 3.5 s. The peak lateral errors at both sharp turns remain below ±0.015 m, indicating superior stability and responsiveness. Notably, after the second turn, GPSO-MPC restores the lateral error to within ±0.01 m in just 4.2 s—a 47.5% reduction in recovery time compared to conventional MPC. These findings further validate the robustness and effectiveness of GPSO-MPC in handling abrupt trajectory variations under dynamic conditions.

Figure 11b illustrates the dynamic response of heading angle error for the three control strategies during the double-lane-shift trajectory tracking task. In the initial simulation phase, all controllers exhibit a rising trend in heading error. Specifically, the conventional MPC reaches a maximum deviation of approximately +2° around 4 s, followed by pronounced bidirectional oscillations between 5 s and 25 s, with a maximum error of −1.5°, indicating poor stability and slow regulation. The PSO-MPC controller records a moderate initial peak error of +1.2° and, despite experiencing two undershoot phases, demonstrates faster convergence than MPC with significantly reduced oscillation amplitude. In contrast, the GPSO-MPC controller consistently achieves the best heading error performance throughout the process. Given its smallest initial error (around 0.5°), it rapidly declines and remains confined within a narrow ±0.3° range. Notably, during the second trajectory disturbance, GPSO-MPC exhibits the smallest rebound amplitude and the smoothest error profile. These results underscore the superior robustness of GPSO-MPC in attitude control under rapid directional changes in nonlinear trajectories.

Figure 12 presents a comparative analysis of tracking output control for double-lane change trajectories. Figure 12a shows the linear velocity profiles of the three control strategies during the double-lane-shift trajectory tracking task. The conventional MPC controller exhibits pronounced velocity fluctuations at multiple turning segments, particularly during the sharp transitions between 5–7 s and 18–22 s, where deviations exceed ± 0.22 m/s. Moreover, the velocity recovery is comparatively slow, indicating noticeable hysteresis. The PSO-MPC strategy reduces the fluctuation amplitude to within ±0.13 m/s, reflecting improved stability; however, oscillations persist in regions with abrupt trajectory changes. In contrast, the GPSO-MPC controller delivers the most stable velocity performance, maintaining variations within ± 0.1 m/s throughout the trajectory. It avoids prominent spikes, even during sudden path transitions, underscoring its superior control, smoothness, and dynamic adaptability.

Figure 12b depicts the angular velocity control outputs of the three strategies. The conventional MPC controller shows substantial fluctuations, with multiple abrupt rises and drops occurring within sharp turning intervals between 5–7 s and 18–21 s. The maximum deviation approaches ± 0.18 rad/s, indicating limited stability in responding to rapid trajectory changes. The PSO-MPC method improves smoothness, restricting peak angular velocity fluctuations to approximately ± 0.12 rad/s and significantly reducing amplitude variations; however, minor discontinuities persist in turning regions. In contrast, the GPSO-MPC controller demonstrates the most stable performance, with a continuous and smooth angular velocity profile devoid of noticeable spikes. Especially in segments with rapid curvature variations, GPSO-MPC ensures seamless transitions, confirming enhanced attitude regulation and robust control capability.

Table 7 presents a comparative analysis of three control algorithms for the double-lane change trajectory tracking task. The results show that GPSO-MPC significantly outperforms both PSO-MPC and conventional MPC in tracking accuracy and stability. Compared to conventional MPC, GPSO-MPC reduces the mean lateral error by 83.7%, from 0.003217 m to 0.0005238 m, and lowers the RMSE lateral error by 52.0% to 0.007826 m, enhancing trajectory tracking precision. For heading angle control, GPSO-MPC decreases the mean error by 75.6%, achieving 0.01863° compared to MPC’s 0.07652°, and reduces the RMSE by 25.4% to 0.3826°, improving stability. In terms of speed control, GPSO-MPC records a mean linear velocity of 2.0185 m/s, closer to the 2 m/s target than MPC’s 2.0643 m/s, reducing deviation by 2.2%. Furthermore, GPSO-MPC reduces the mean angular velocity deviation from zero by 72.1%, reaching −0.003217 rad/s. Overall, GPSO-MPC demonstrates notable improvements over both MPC and PSO-MPC, offering superior tracking accuracy, stability, and adaptability, thereby confirming its effectiveness for high-precision path tracking in dynamic environments.

## 7. Conclusions

Motivated by the limitations of conventional MPC in wheeled mobile robot trajectory tracking—particularly its dependence on empirically tuned parameters and limited adaptability to dynamic environments—this study proposes a GPSO-MPC control strategy that integrates GWO with an enhanced PSO algorithm. The theoretical framework, algorithmic innovations, and simulation validations lead to the following key findings:

1. A hybrid GPSO algorithm is developed by integrating the hierarchical search mechanism of GWO with a chaos-perturbed adaptive PSO. This design enhances global exploration and prevents premature convergence. A multi-objective fitness function is constructed to balance tracking accuracy, control smoothness, and constraint feasibility.
2. Based on a four-wheeled differential-drive kinematic model of an underwater mobile robot, an MPC controller is implemented using rolling optimization and explicit constraint handling. The GPSO algorithm enables real-time adaptive tuning of key MPC parameters (Np, Nc, Q, R), improving robustness under nonlinear conditions.
3. Simulation results demonstrate that GPSO-MPC significantly improves tracking accuracy and stability over conventional MPC and PSO-MPC. In double-lane change trajectories, GPSO-MPC reduces mean lateral error by 83.7%, heading error by 75.6%, and velocity deviation by 2.2%. For circular trajectories, it reduces mean lateral error by 45.97% and RMSE lateral error by 43.63%, while reducing mean heading angle error by 23.52% and cutting RMSE heading angle error by 24.63% relative to PSO-MPC. Additionally, GPSO-MPC lowers velocity deviation by 7.13% compared to MPC in circular paths and keeps mean angular velocity only 2.37% below the target. Overall, GPSO-MPC consistently outperformed both PSO-MPC and conventional MPC in tracking precision and control stability across trajectory types.

In summary, the GPSO-MPC controller significantly enhances trajectory tracking accuracy, heading regulation, and input stability. Its rapid response, high steady-state precision, and strong adaptability make it a promising solution for mobile robots operating in complex and dynamically changing environments.

## Figures and Tables

**Table 1:** Nomenclature of Robot Kinematic Model on Soft Slope.

Parameters	Definition	Unit
*v*	Linear velocity of the robot	m/s
*v_i_*	Linear velocity of the *i*-th wheel of the robot	m/s
*v_x_*	Longitudinal velocity of the robot	m/s
*v_y_*	Lateral velocity of the robot	m/s
*w*	Steering angular velocity of the robot	rad/s
*w_i_*	Angular velocity of the *i*-th wheel of the robot	rad/s
*β*	Side-slip angle at the robot’s center of mass	rad
*β_i_*	Side-slip angle of the *i*-th wheel of the robot	rad
*θ*	Heading angle	rad
*r*	Tire radius	m
*b*	Tire width	m
*D*	Distance from front–rear wheel axles to robot axis	m
*L*	Distance from left–right wheel axles to robot axis	m
*α*	Slope angle	rad

**Table 2:** Seabed Soil Parameters.

Parameters	Value	Unit
*c*_1_	0.82	
*c*_2_	1.18	
*c*	3.6	kPa
*k_j_*	175	m
*k_c_*	35	kN^n+1^
*φ*	28	°
*k_φ_*	1000	kN^n+2^
*n*	0.6	

**Table 3:** Nomenclature of Robot Dynamic Model on Soft Slope.

Parameters	Definition	Unit
*G*	Gravitational force of the robot	N
*W_i_*	Normal load of the *i*-th wheel	N
*F_f_*	Buoyancy force	N
*F_W_*	Water resistance force	N
*F_ti_*	Traction force of the *i*-th wheel	N
*F_ri_*	Ground resistance force on the *i*-th wheel	N
*F_yi_*	Lateral force of the *i*-th wheel	N
*Iz*	Moment of inertia of the robot	kg·m^2^
*m*	Mass of the robot	kg
*T_i_*	Wheel driving torque	N·m
*h*	Mass center height of the robot	m
*c_β_*	The wheel cornering stiffness	
*μ*	Ground friction coefficient	
*ρ*	Seawater density	kg/m^3^
*C_d_*	The drag coefficient	

**Table 4:** Robot Technical Parameters.

Parameters	Definition	Value/(Unit)
*m*	Robot mass	720/(kg)
*F_f_*	Robot buoyancy	5037/(N)
*D*	Distance from front–rear wheel axles to robot axis	0.795/(m)
*L*	Distance from left–right wheel axles to robot axis	0.47/(m)
*r*	Tire radius	0.165/(m)
*b*	Tire width	0.11/(m)
*T_max_*	Torque of the drive-wheel motor	120 (N/s)
*h*	Mass center height of the robot	0.32/(m)
*Iz*	Moment inertia of the robot	32/(kg·m^2^)
*μ*	Ground friction coefficient	0.28
*c_β_*	The wheel relative cornering stiffness	0.22
*ρ*	Seawater density	1025 (kg/m^3^)
*C_d_*	The drag coefficient	

**Table 5:** Configuration of MPC Controller Parameters.

Parameters	Definition	Value/(Unit)
*N_P_*	Prediction horizon	15
*N_C_*	Control horizon	5
*Q*	State error weight	diag[1.5,1.0,2.5]
*R*	Control input weight	diag[0.05,0.1]
*dt*	Sampling time	0.1/(s)
*M*	Number of particles	40
*K*	Maximum number of iterations	35
*ω_max_*	Maximum inertia weight	0.9
*ω_min_*	Minimum inertia weight	0.4
*C*_1_, *C*_2_, *C*_3_	Initial learning factor value	2.0
*e_y,max_*	Maximum lateral error	0.2/(m)
*e* *_θ_* *_,max_*	Maximum heading Angle error	2/(°)

**Table 6:** Comparison of different performance metrics in circular trajectories.

Performance Metric	MPC	PSO-MPC	GPSO-MPC
Mean lateral error (m)	0.14028	0.10433	0.075793
Root-mean-square lateral error (m)	0.14658	0.10888	0.08263
Mean heading angle error (°)	1.0022	0.58865	0.45022
Root-mean-square heading angle error (°)	1.1417	0.76093	0.57349
Mean linear velocity (m/s)	1.5817	1.4252	1.469
Mean angular velocity (rad/s)	0.30701	0.28768	0.29288

**Table 7:** Comparison of different performance metrics in Double-Lane Change trajectories.

Performance Metric	MPC	PSO-MPC	GPSO-MPC
Mean lateral error (m)	−0.0065286	−0.0015373	0.00030023
Root-mean-square lateral error (m)	0.026333	0.013869	0.0093388
Mean heading angle error (°)	0.11315	0.084	0.028881
Root-mean-square heading angle error (°)	0.6859	0.46362	0.32083
Mean linear velocity (m/s)	2.0837	2.0334	2.0257
Mean angular velocity (rad/s)	−0.014764	−0.010382	−0.004068

## Data Availability

Data are contained within the article.
